# PROPEL: implementation of an evidence based pelvic floor muscle training intervention for women with pelvic organ prolapse: a realist evaluation and outcomes study protocol

**DOI:** 10.1186/s12913-017-2795-x

**Published:** 2017-12-22

**Authors:** Margaret Maxwell, Karen Semple, Sarah Wane, Andrew Elders, Edward Duncan, Purva Abhyankar, Joyce Wilkinson, Douglas Tincello, Eileen Calveley, Mary MacFarlane, Doreen McClurg, Karen Guerrero, Helen Mason, Suzanne Hagen

**Affiliations:** 1 Nursing, Midwifery and Allied Health Professionals Research Unit, University of Stirling, Stirling, UK; 2 Glasgow Caledonian University, Glasgow, UK; 3 University of Leicester, Leicester, UK; 4 NHS Greater Glasgow and Clyde, Glasgow, UK

**Keywords:** Pelvic organ prolapse, Pelvic floor muscle training, Implementation

## Abstract

**Background:**

Pelvic Organ Prolapse (POP) is estimated to affect 41%–50% of women aged over 40. Findings from the multi-centre randomised controlled “Pelvic Organ Prolapse PhysiotherapY” (POPPY) trial showed that individualised pelvic floor muscle training (PFMT) was effective in reducing symptoms of prolapse, improved quality of life and showed clear potential to be cost-effective. However, provision of PFMT for prolapse continues to vary across the UK, with limited numbers of women’s health physiotherapists specialising in its delivery. Implementation of this robust evidence from the POPPY trial will require attention to different models of delivery (e.g. staff skill mix) to fit with differing care environments.

**Methods:**

A Realist Evaluation (RE) of implementation and outcomes of PFMT delivery in contrasting NHS settings will be conducted using multiple case study sites. Involving substantial local stakeholder engagement will permit a detailed exploration of how local sites make decisions on how to deliver PFMT and how these lead to service change. The RE will track how implementation is working; identify what influences outcomes; and, guided by the RE-AIM framework, will collect robust outcomes data. This will require mixed methods data collection and analysis.

Qualitative data will be collected at four time-points across each site to understand local contexts and decisions regarding options for intervention delivery and to monitor implementation, uptake, adherence and outcomes. Patient outcome data will be collected at baseline, six months and one year follow-up for 120 women. Primary outcome will be the Pelvic Organ Prolapse Symptom Score (POP-SS). An economic evaluation will assess the costs and benefits associated with different delivery models taking account of further health care resource use by the women. Cost data will be combined with the primary outcome in a cost effectiveness analysis, and the EQ-5D-5L data in a cost utility analysis for each of the different models of delivery.

**Discussion:**

Study of the implementation of varying models of service delivery of PFMT across contrasting sites combined with outcomes data and a cost effectiveness analysis will provide insight into the implementation and value of different models of PFMT service delivery and the cost benefits to the NHS in the longer term.

## Background

Pelvic organ prolapse (POP) is a common condition affecting 41%–50% of women over the age of 40 [1, 2]. POP is defined as the symptomatic descent of one or more of the anterior or posterior vaginal walls, apex of the vagina or the uterus (cervix) [3]. A rising number of POP cases result in surgery (15 to 49 cases per 10,000 women-years) [4–6]. As well as having associated morbidity, surgery is often prone to failure; around 30% of women undergo repeat operations, and the time intervals between repeat procedures shorten with each successive repair [5]. In addition, repair of one type of prolapse may predispose the woman to the development of a different type of prolapse in another compartment of the vagina [5]. There remain concerns surrounding the use of synthetic mesh, one option for prolapse surgery. Mesh-related complications are frequently reported, with up to a 35% removal rate [7] resulting in the Chief Medical Officer in Scotland (UK) requesting that all Health Boards consider suspending routine use of polypropylene mesh implants for the management of pelvic organ prolapse and incontinence in June 2014 [8].

Given these concerns, there is an ever more pressing need for evidence-based non-surgical options to be made available to women [9]. A recent definitive trial has shown that pelvic floor muscle training is an effective and potentially cost effective treatment and should be recommended as a first line treatment for POP [10]. The Pelvic Organ Prolapse PhysiotherapY (POPPY) trial was a multi-centre randomised controlled trial of the effectiveness and cost-effectiveness of individualised pelvic floor muscle training (PFMT) delivered by specialist physiotherapists compared to a lifestyle advice leaflet in newly diagnosed women with symptomatic stage I, II or III prolapse [10]. POPPY constitutes the largest, most rigorous, pragmatic trial of PFMT for prolapse, and as such provides the necessary evidence to inform future practice. Individualised PFMT was found to be effective in reducing women’s symptoms of prolapse, and providing perceived improvements in prolapse and related quality of life. The PFMT intervention also shows potential to be a cost-effective treatment.

However, knowledge of efficacy and effectiveness is not enough to ensure implementation of this evidence into practice. PFMT provision varies across the UK as there is limited availability of specialist physiotherapy services to meet the known levels of demand [11, 12]. Delivery methods that can enhance service capacity and increase availability and choice for women are required. Such delivery methods also need to be tested to ensure that the outcomes achieved under trial conditions are maintained.

This study aims to maximise the delivery of effective PFMT for women with POP through the study of its implementation in a number of diverse settings using an evidence-based PFMT protocol. This will involve developing different service delivery models, incorporating a variety of staff skill mixes and sessions to increase capacity, with the format of delivery being determined locally. These will be addressed through the following specific study aims:

- To explore the potential for different groups of staff skill mix to deliver PFMT without compromising the achievement of clinical outcomes
- To explore fidelity or variation to PFMT protocol (e.g. number and type of sessions) and the impact of any variations
- To establish the levels of support required by non-specialist physiotherapists to deliver PFMT
- To explore the acceptability and outcomes for women of different delivery models
- To establish the costs and benefits associated with each model of delivery
- To contribute to knowledge of how and why implementation processes are successful (or not) through exploring what works, for whom and in what circumstances.

Implementation Science is an emerging field aiming to foster understanding of the complex and multi-level processes involved in the uptake and spread/scale-up of evidence-based interventions and programmes [13]. It aims not only to reduce a gap in quality of care, but also to advance the science of implementation by providing generalisable knowledge that will be useful for other settings and contexts. It can help to identify barriers to implementation but should also extend this to understand how and why implementation processes are effective [14]. To do this we need to study implementation strategies and the contexts and processes in which implementation strategies are delivered. Such research can advance the adoption, sustainability and wider roll out of cost-effective evidence based interventions and are a necessary step in the MRC’s evaluation of complex interventions framework [15].

## Methods/design

### Study design

The study will involve: A) A Realist Evaluation (RE) using case studies of implementation of PFMT delivery in three varying NHS settings; B) An outcomes study of PFMT delivery in the different models; C) An economic evaluation to compare the costs and benefits of the different models.

### A) Realist Evaluation using case studies of implementation

The RE will involve substantial local stakeholder engagement, allowing local sites to make decisions on how to deliver PFMT (within their resources) and will elicit local folk theories around: how implementation is supposed to work; track how the implementation is working (including fidelity to the PFMT protocol); and understand what influences outcomes.

### Setting

The Realist Evaluation will be based at three NHS sites in the UK with either specialist pelvic floor or women’s health services, and selecting sites reflecting a mix of urban/rural locations, previous involvement/non-involvement in POPPY and current differences in service delivery models (see Table 1). A lead specialist physiotherapist based at each location will facilitate liaising with local service managers/consultants and staff to take part in developing a local model of PFMT service provision; the research team will consent those participating into the study.

**Table 1:** Realist Evaluation Study site descriptions

Site	Urban/rural	POPPY experience	Current dominant model
1	Urban	POPPY recruiting centre	Primary and secondary care provision of specialist physiotherapy referred by primary care and acute services with a mix of 1:1 and group provision available. Women not routinely sent to physiotherapy as first line treatment. Several POPPY physiotherapists providing current input to women with POP
2	Urban	Large involvement in medically driven trials but no focus on physiotherapy based trials	Currently has some specialist physiotherapy involvement but greater specialist nurse-led service for women
		No POPPY involvement	
3	Rural	No POPPY involvement and limited other trial involvement	Current interest in re-design and expanding to junior grade physiotherapists and other nursing staff (as practitioners with special interest)

*POP* Pelvic Organ Prolapse, *POPPY* Pelvic Organ Prolapse PhysiotherapY trial

### B) Outcomes study

Outcomes data will be collected before and after women receive the PFMT intervention in each location/model of service delivery to establish whether the improvements observed in the POPPY trial are also observed under different models of delivery and in non-trial real world NHS settings (see Table 2).

**Table 2:** Outcomes Study site descriptions

Site	Urban/rural	POPPY experience	Current dominant model
1	Urban	POPPY recruiting centre	Primary and secondary care provision of specialist physiotherapy referred by primary care and acute services with a mix of 1:1 and group provision available. Women not routinely sent to physiotherapy as first line treatment. Several POPPY physiotherapists providing current input to women with POP
2	Urban	Large involvement in medically driven trials but no focus on physiotherapy based trials	Currently has some specialist physiotherapy involvement but greater specialist nurse-led service for women
		No POPPY involvement	
3	Rural	No POPPY involvement and limited other trial involvement	Current interest in re-design and expanding to junior grade physiotherapists and other nursing staff (as practitioners with special interest)
4	Urban	No POPPY involvement	Currently has some specialist physiotherapy involvement, looking to increase capacity for PFMT provision with other staff groups
5	Urban	No POPPY involvement	Currently has some specialist physiotherapy involvement, looking to increase capacity for PFMT provision with other staff groups

*PFMT* Pelvic Floor Muscle Training, *POP* Pelvic Organ Prolapse, *POPPY* Pelvic Organ Prolapse PhysiotherapY trial

### Recruitment/sampling

Women who are awaiting treatment for prolapse at study sites will be potential recruits if they are eligible and provide written, informed consent to take part. We aim to recruit 120 women to the outcomes study.

### Inclusion criteria

- Women 18 years of age or above, presenting with symptomatic stage I, II or III prolapse of any type who are suitable to be referred for PFMT and who are willing to take part in the research and comply with necessary data collection.

### Exclusion criteria

- Women who are pregnant or less than one year postnatal
- Women who have prolapse greater than stage III, who have pelvic cancer, cognitive impairment or neurological disease (as indicated by referral source or patient/carer reported at assessment consultation)

### Sample size

We have assumed that the minimum clinically important difference in the POP − SS is 2 [16] and that the standard deviation of the differences in POP − SS between baseline and one year will be 5.5 (as observed in POPPY). A sample size of 120 paired observations (with 2-sided alpha = 0.05) will therefore provide 80% power to detect important differences in POP − SS between baseline and one year.

### Setting

The outcomes study will be based at five sites, those three taking part in the RE plus two further sites who will be recruited to specifically take part in the outcomes study (see Table 1). Clinicians identified locally at each site will be trained to deliver the PFMT intervention by Pelvic, Obstetric and Gynaecological Physiotherapy approved trainers.

### C) Economic evaluation

An economic evaluation will compare costs and outcomes of the different models of service delivery.

## The PFMT intervention

PFMT refers to the regular practice of repetitive pelvic floor muscle contractions in order to produce a training effect on the muscles. The aim of a PFMT programme is to increase the strength of the muscles to build up muscle volume thereby improving structural support, increase endurance, improve resting tone, improve muscle fibre recruitment through improved nerve function and properties of muscle fibres; and improve cognitive awareness of body posture and a relaxed versus an un-relaxed state of the pelvic floor [17]. To produce improvements in muscle strength and endurance the basic physiological principles must be adhered to [18]. These include: overload (muscles need to perform more work than usual resulting in fatigue); specificity (muscles must be trained with physical activity that replicates as closely as possible the functional movement required); maintenance and reversibility (benefits of the exercise are reversible if they are not undertaken on a regular basis). Evidence suggests that for effective strength training in skeletal muscles in adults, three to five sets of 8 to 12 slow velocity, close to maximal contractions per day should be performed 2 to 5 days per week for 16 weeks [19].

The PFMT protocol will normally be delivered during five therapy appointments at weeks 0, 2, 6, 11, 16 with fewer or more appointments as judged by therapists based on client needs. The intervention will normally last 16 weeks. Each of the five appointments will take around 30 min each, with the initial appointment expected to take longer (45–60 min). The initial assessment appointment will elicit and record basic demographic and medical history along with a visual and digital assessment of the vagina and pelvic floor muscles. The initial assessment aims to provide accurate knowledge of the condition of the perineum and vagina, the woman’s capacity for contracting and relaxing her pelvic floor muscles and the influence of muscles on any visible or palpable prolapse. This information is recorded using the Oxford Classification (PERFECT scheme) and provides a basis for determining the dose/frequency of PFMT [20].

Women are taught the correct exercise technique and this is confirmed on digital palpation by the therapist with the woman in the supine position. Women are encouraged to become aware of contracting and holding the muscles, and also of relaxing them. This is facilitated by the use of different exercise positions (e.g. side lying), counter pressure on their perineum, coordination of contraction/relaxation with breathing. Women are taught to counterbrace, that is, to pre-contract their pelvic floor muscles prior to an increase in intra-abdominal pressure (e.g. coughing, sneezing, lifting), a technique known as “the knack” [21].

The initial exercise programme is identified and agreed between the woman and therapist over the first and second appointments, according to the woman’s ability. The exercise programme is practised during subsequent appointments to allow the therapist to assess progress and adjust the programme as necessary. Home exercise may be prescribed from the first appointment, or as soon as the therapist confirms a correct technique has been achieved.

Home exercise is tailored to the individual woman, ensuring a training effect on the muscles is achieved, but that the programme is manageable for the woman. Targets of maximum strength, endurance and repetitions are set, aiming towards a programme of three to five sets per day of 10 maximum strength pelvic floor muscle contractions, holding each contraction for up to 10 s with a 4 s rest between contractions, followed by up to 50 fast contractions. Exercise position is varied (lying, sitting, standing, squatting). Potential for progression is determined during the therapy appointments and is carried over to home exercise. Progression is tailored for each woman [18] and consists of increased number of contractions, increased length of hold, decreased rest periods and the introduction of more difficult positions. Therapists will make use of appropriate motivational techniques and advice detailed within the protocol to encourage correct exercise technique and adherence to the prescribed programme. Women with urinary and/or bowel symptoms including urgency, frequency, incontinence and defecation difficulties will be given advice on to improve these symptoms.

Vaginal examinations will be undertaken following Chartered Society of Physiotherapy and Royal College of Obstetrics and Gynaecology guidelines for performing intimate examinations. Local infection policy will be adhered to and the woman’s informed consent will be obtained. A chaperone will be offered. Repeat pelvic floor muscle assessments will be undertaken at each session to assess progress and modify home exercises if the woman is agreeable.

## Training to deliver the PFMT intervention

**Fig. 1:**
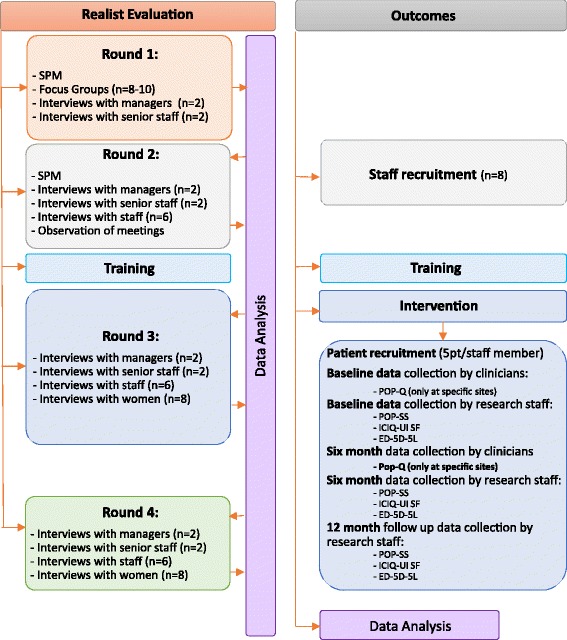
PROPEL Realist Evaluation and outcomes study flowchart EQ-5D-5 L: EuroQol 5 dimensions questionnaire; ICIQ-UI SF: International Consultation on Incontinence Questionnaire Urinary Incontinence short form; n: number of participants; PFMT: Pelvic floor muscle training; POP: Pelvic organ prolapse

Training will be delivered after Round 2 data collection has been completed. Clinicians who have been identified locally through the service planning process will undergo one full day of bespoke training to prepare them for delivering PFMT to women with POP recruited to the study. This training has been developed specifically for this study in partnership with the Pelvic, Obstetric and Gynaecological Physiotherapy (POGP) Group. The training will be delivered by two POGP trainers per full day session and one member of the research team. It includes training in PROPEL specific research processes and paperwork, an introduction to internal examination, and delivery of the PFMT programme for women. This training will ensure that they meet minimum levels of competencies developed in agreement with the POGP. There will also be ongoing support available locally for staff who have received this training from specialist women’s health physiotherapists, including observation of initial assessment and treatment. Any additional support or training required by staff to deliver the PFMT programme for women will be recorded (Fig. 1).

## Data collection

### A) Realist Evaluation

Qualitative data for the RE will be collected in four rounds at different time-points over an 18 month implementation period across each site to understand local contexts and decisions regarding delivery of PFMT for prolapse and to monitor implementation, up-take, adherence and impact. **Error! Reference source not found.** Summarises data to be collected during each round. Two rounds of data collection will take place during the planning stages (what to implement and how, and local theories of change), one round during implementation/delivery of the models (how is it working) and one round immediately post implementation (reflection on how it has worked). All focus groups, interviews and service planning meetings will be recorded and transcribed.

### B) Outcomes study

It is anticipated that each staff member in the five sites will recruit five women to take part in the study and who will: agree to complete study outcome questionnaires, have the POP-Q assessment (pre- and 6 months post) and take part in qualitative interviews if requested. Patient outcomes as used in the POPPY trial will be collected at baseline (immediately prior to first (assessment) appointment) and at six months and one year follow-up; clinicians will collect POP-Q data at baseline and 6 months during specified appointments and the remaining measures (POP-SS, EQ-5D-5 L, ICIQ-UI SF and patient demographic data all collated within patient completed questionnaire) will be collected by post at each time point.

### C) Economic Evaluation

Resource use for the PFMT will be collected. Resource use will be recorded prospectively for every woman within the study. For the PFMT intervention, cost details will be gathered from the Intervention Therapists, recorded at the time of intervention (e.g. length of appointments, materials used). Data on the use of primary and secondary care services by the women, including medications, GP visits and uptake of surgery, will be collected using participant questionnaires devised by the research team which will be administered at each of the follow up time periods. Unit costs/prices for resource use will be obtained using published estimates for health care services and/or interventions. Costs to the women will be collected in the participant questionnaires. Data will be collected on the mode of travel, cost of travel if applicable, amount of time the women take out of their usual activities such as work, to go to the appointments and similar data from family members that either accompany them or look after their children.

To measure health outcomes, the EQ-5D-5 L generic instrument will be used [22]. This instrument will provide the quality of life weights (utility values) to compute quality adjusted life years (QALYs). Study participants will complete the EQ-5D-5 L generic instrument within the questionnaires at baseline and at 6, and 12 months after randomisation.

## Outcome measures

The primary outcome, the Pelvic Organ Prolapse Symptom Score (POP-SS), will be assessed at baseline, 6 months and 12 months via a self-completed questionnaire. The POP-SS is a validated tool that includes seven items relating to the frequency of prolapse symptoms over the previous four weeks. Each item is scored from 0 (never) to 4 (all of the time) [23]. This questionnaire booklet will also measure secondary outcomes, assessing quality of life with the use of the EQ-5D-5L [24]; urinary incontinence with the ICIQ-UI short-form [25]; and service related outcomes including the need for further treatment. Six and 12 month questionnaires will also address the women’s adherence to the advice they have been given throughout the intervention (i.e. avoiding heavy lifting, changes to diet, weight loss) and also detail any further medical interventions that have been or are expected to be completed.

### POP-Q

Prolapse severity will be assessed as a secondary outcome with the use of the Pelvic Organ Prolapse Quantification system (POP-Q) [26]; a standardised and internationally recognised quantification system for prolapse. It is an objective, site-specific system for describing, quantifying and staging pelvic support in women. POP-Q will be performed for each woman in the outcomes study either by a consultant gynaecologist or a specialist women’s health physiotherapist trained to perform this assessment.

## Data analysis

### A) Realist Evaluation

Data from each round will be transcribed verbatim, organised using the data management software NVivo version 11, and analysed using a thematic analysis approach (Ritchie and Spencer [27]). Following familiarisation with the data by a minimum of two researchers, a thematic framework will be developed and agreed using data from a subsample of interview transcripts and the core concepts of the realist approach – contexts, mechanisms and outcomes. This framework will then be applied across the dataset through indexing of data segments. Indexing will be conducted by two researchers who will cross check a 10% sample of transcripts. Data will then be tabulated and conceptual maps used to make links between themes.

Data in each round will be summarised and synthesised across three sites to articulate the context (C) and mechanisms (M) of action and relate these to observed (quantitative and qualitative) outcomes (O) – leading to the development of CMO configurations. Round 1 and 2 data will be used to generate hypotheses about what potential mechanisms could or would be generated by the implementation, in what contexts, to achieve what outcomes. Round 3 data will be used to test these conjectured CMO configurations to understand what mechanisms were actually triggered in practice and how they were enabled or constricted by certain contexts. Finally, the hypothesised and observed CMOs will be used to test if they explain the complex patterning of outcomes of the PFMT implementation (qualitative and quantitative) gathered in round 4 and the outcomes study.

### B) Outcomes data

Descriptive statistics will be tabulated for all outcomes at each time point, showing means and SDs for POP − SS and EQ − 5D-5 L, proportions for POP − Q, the need for further treatment, and frequencies for the number of NHS contacts. Differences (and correlations where appropriate) between baseline and 6 − months and between baseline and 12 − months will be calculated. We will initially examine the differences before and after intervention using paired t-tests and chi-square tests. We will fit appropriate generalised linear models on the difference between outcomes before and after intervention, adjusting for baseline measurements and potential confounders (e.g. demographic factors, study site). If possible, we will also explore the effect of different levels of adherence to PFMT using structural mean models [28]. Complete case analyses will be performed throughout, with missing data being addressed in secondary analyses using multiple imputation methods.

POP − SS and EQ − 5D 5L will be treated as continuous data, POP − Q as ordinal, NHS contacts as count data and the need for further treatment (e.g. surgery or pessary) as binary data. In order to facilitate the understanding of prolapse related outcomes, individual items of the POP − SS will also be analysed separately and an additional dichotomous POP − Q measure (above/below the hymen) will be included in the analysis.

### C) Economic evaluation

Incremental cost-effectiveness ratios (ICERs) will be computed comparing the costs and outcomes of the different modes of delivery. The difference in effectiveness will be expressed in terms of the change in POP-SS Score. These data will be retrieved from the women’s questionnaire responses to the POP-SS outcome measure. The difference in utility will be expressed in terms of QALYs. These data will come from the EQ-5D-5L instrument. Where appropriate the analysis of incremental costs, effectiveness and cost-effectiveness will be based on similar statistical models as those already outlined (Table 3).

**Table 3:** PROPEL Realist Evaluation data collection process

Data collection method	Participants (per site)	Round 1 - Discussion and development of the models	Round 2 - Operationalising the models for successful implementation	Round 3 - Delivering the models	Round 4 - Review of the models
Focus groups with women	8–10 women living with prolapse who have been through the current service, identified by specialist PTs in the area	To understand initial perceptions of the acceptability of PFMT, preferences for service delivery models, what the service might look like (referrals, location etc.)			
Service planning meeting	Local service managers, clinical leads, consultants and other relevant staff groups identified by the study specialist PTs at each location and invited to attend	(1 meeting/site) Discuss current service provision, local capacity issues and how these might be addressed with the available or an extended staff pool	(1 or 2 meetings/site) Observe the decision making process, firming planning decisions around what to implement and how the service will be operationalised within the current service structure		
Interviews with managers/service leads	2 interviewees selected from key decision makers, likely those attending the SPM	To explore any anticipated barriers or facilitators and explore further contextual detail that may influence choice of service model and implementation	To explore operationalisation of the decision and how this will be translated to staff involved in the project	To understand how referrals are made and cascaded to trained staff, any anxieties voiced about training/support, local resources	To explore the perceptions of the success of the model and sustainability, possible modifications, impact of the study, future plans
Interviews with consultants/senior AHPs/senior nurses/GPs	2 interviewees selected from key decision makers, likely those attending SPM	To explore their perceptions of PFMT delivery and the proposed models and any barriers of facilitators they anticipate	To explore how they see their involvement in the service change	To discuss any problems they have observed during implementation and any impact they perceive the PFMT services are having	To explore the perceptions of the implementation, its impact and future (should it be sustained/expanded)
Interviews with staff delivering PFMT	6 staff who are delivering PFMT within the new service		To explore what they think of the decision to have them deliver PFMT, their expectations of training and delivery and any anticipated impact on staff	To discuss how they feel the implementation is going, any concern or problems in delivery/referrals, their perceived early impact of delivering PFMT	To discuss the overall experiences of delivering PFMT, perceived impact of their role, anything done difference, key drivers to success
Interviews with a sample of women	8 women who have been recruited as patients into the new service, representing women seen by a range of staff mixes utilised locally			To discuss their experiences of POP, expectations of treatment and any perceived issues for compliance	To explore their experience of the intervention, adherence to appointments and therapy, and outcomes

*PFMT* Pelvic floor muscle training, *POP* Pelvic organ prolapse, *PTs* Physiotherapists, *SPM* Service planning meeting

## Discussion

This study will enhance knowledge concerning implementation of the evidence based PFMT protocol in different NHS settings, potentially using different staff/skill mixes, intensity of delivery and other innovations to increase service capacity. The definitive POPPY trial did not investigate barriers or facilitators to uptake and adoption of PFMT across varied NHS settings. This study will provide such knowledge to bridge the ‘implementation gap’. We have combined a study of implementation alongside a robust outcomes study, ensuring that we can provide robust evidence of what works for whom and in what circumstances. The patient level outcomes data will also ensure that we can deliver a robust economic evaluation.

The mixed methods approach being employed in the PROPEL study will provide valuable data on the feasibility of delivering this intervention using a number of models, the resources and environment needed to make these work and the efficacy of using different clinical groups than those studied in the POPPY trial.

We have worked in partnership with the POGP to develop bespoke training for a variety of clinicians who might be identified in different health care settings to deliver individualised pelvic floor muscle training to women presenting with stage I, II or III pelvic organ prolapse. This training has been developed with the intention of providing clinicians with minimum competencies in undertaking internal vaginal examinations and providing appropriate education for clinicians to provide individualised education and PFMT to women with POP.

## Dissemination and outputs

Two “Dissemination for Implementation” Workshops will be held: one in England and one in Scotland. These workshops will bring together clinical leads, service managers, clinicians, professional network organisations (such as POGP), PPI partners and other key stakeholders, from the included sites and other NHS Trusts, to hear and discuss study findings. These events will also ask NHS service-based participants to discuss the relevance of findings to their local area and to consider and plan for local implementation. This can be achieved by taking on board the knowledge for ‘mechanisms’ of action that lead to successful implementation, alongside evidence of what works for who, in what circumstances, and also alongside knowledge of longer term outcomes. For example, if benefits are maintained then ‘savings’ could provide a strong argument for investment in PFMT. If benefits are not maintained then it might be that ‘refresher’ sessions need to be built into future delivery. These will consist of information sharing but also include an ‘action planning’ component for service managers and those responsible for the delivery of non-surgical interventions for POP to begin to plan for local service re-design that can deliver PFMT to larger numbers of women. These events will also act as ‘data gathering’ opportunities for the study, which is why they are included as a study objective: gathering data on potential opposition and barriers to implementation, how services make sense of and use our study findings and apply these to their own services. We expect the key people who lead and manage services for women with POP to attend and to leave with an action plan.

The findings of the study will also be disseminated through a number of specialist bodies, responsible for guiding clinical practice and policy, research priorities, governance and training of professionals (e.g. the International Continence Society (ICS), the Pelvic, Obstetric and Gynaecological Physiotherapists group (POGP), the Association for Continence Advice (ACA), the British Society of Urogynaecologists (BSUG) and the Royal College of Obstetrics and Gynaecology (RCOG)) and relevant patient and public involvement organisations (e.g. Bladder and Bowel UK, CSP website).

## Abbreviations

EQ-5D-5L: EuroQol 5 dimensions questionnaire
ICIQ-UI SF: International Consultation on Incontinence Questionnaire short form
NMAHP RU: Nursing, Midwifery and Allied Health Professions Research Unit
PFMT: Pelvic Floor Muscle Training
POGP: Pelvic, Obstetric and Gynaecological Physiotherapy group
POP: Pelvic Organ Prolapse
POPPY: Pelvic Organ Prolapse PhysiotherapY
POP-Q: Pelvic Organ Prolapse Quantification
POP-SS: Pelvic Organ Prolapse Symptom Score
QALY: Quality Adjusted Life Years
RE: Realist Evaluation
SPM: Service Planning Meeting
